# The Role of Short-Chain Fatty Acids From Gut Microbiota in Gut-Brain Communication

**DOI:** 10.3389/fendo.2020.00025

**Published:** 2020-01-31

**Authors:** Ygor Parladore Silva, Andressa Bernardi, Rudimar Luiz Frozza

**Affiliations:** ^1^Laboratory on Thymus Research, Oswaldo Cruz Institute, Oswaldo Cruz Foundation, Rio de Janeiro, Brazil; ^2^Laboratory of Inflammation, Oswaldo Cruz Institute, Oswaldo Cruz Foundation, Rio de Janeiro, Brazil

**Keywords:** central nervous system, neuroinflammation, gut-brain axis, gut microbiota, short-chain fatty acids

## Abstract

A substantial body of evidence supports that the gut microbiota plays a pivotal role in the regulation of metabolic, endocrine and immune functions. In recent years, there has been growing recognition of the involvement of the gut microbiota in the modulation of multiple neurochemical pathways through the highly interconnected gut-brain axis. Although amazing scientific breakthroughs over the last few years have expanded our knowledge on the communication between microbes and their hosts, the underpinnings of microbiota-gut-brain crosstalk remain to be determined. Short-chain fatty acids (SCFAs), the main metabolites produced in the colon by bacterial fermentation of dietary fibers and resistant starch, are speculated to play a key role in neuro-immunoendocrine regulation. However, the underlying mechanisms through which SCFAs might influence brain physiology and behavior have not been fully elucidated. In this review, we outline the current knowledge about the involvement of SCFAs in microbiota-gut-brain interactions. We also highlight how the development of future treatments for central nervous system (CNS) disorders can take advantage of the intimate and mutual interactions of the gut microbiota with the brain by exploring the role of SCFAs in the regulation of neuro-immunoendocrine function.

## Introduction

The human body is inhabited by a wide variety of commensal microorganisms collectively called the microbiota. This host microbiota colonizes the skin and several mucosal cavities (nasal, oral, pulmonary, and vaginal); however, it is in the gastrointestinal (GI) tract that these organisms reach extraordinary densities since trillions of bacteria, fungi, and viruses coexist in symbiosis with the host for potential mutual benefit (1–3). Despite its significant influence on the state of human health and the development or progression of diseases, it is only in the last 20 years that our gut microbiota has become the focus of intense studies. Therefore, its pivotal roles in protecting against pathogens, regulating metabolic, endocrine, and immune functions and in influencing drug metabolism and absorption have started to be elucidated (4, 5). Further, it was recently unveiled that the influence of the microbiota is not restricted to the GI tract; it plays a major role in the bidirectional communication between the GI tract and the central nervous system (CNS). The growing body of evidence indicating that the gut microbiota exerts a profound influence on key brain processes has led to the development of the microbiota-gut-brain axis concept, which has attracted the interest of researchers worldwide (6–11).

Although the precise mechanisms involved in the crosstalk between the gut microbiota and brain remain to be fully determined, there are a number of potential pathways through which the gut microbiota can influence brain function (9). Microorganisms can influence CNS processes bidirectionally via the vagus nerve (12) and through modulation of the immune system (6), the hypothalamic-pituitary-adrenal (HPA) axis (13, 14), and tryptophan metabolism (15), along with their ability to synthetize a number of neurotransmitters (16–18) and produce metabolites, such as short-chain fatty acids (SCFAs), that possess neuroactive properties (17, 19–21).

The SCFAs acetate, propionate, and butyrate are the main metabolites produced in the colon by bacterial fermentation of dietary fibers and resistant starch (22). In addition to the long-known role of the colon in energy supply and trophic factors (22), as well as the regulation of T regulatory (Treg) cell colonies (23, 24), growing evidence supports the idea that SCFAs also exert crucial physiological effects on several organs, including the brain (17, 20, 21). This hypothesis is supported by studies in animals and humans showing that gut microbiota dysbiosis has been implicated in behavioral and neurologic pathologies, such as depression, Alzheimer's (AD) and Parkinson's (PD) diseases and autism spectrum disorder (ASD) (9, 21, 25–27). Furthermore, microbiota manipulation and SCFA administration have been proposed as treatment targets for such diseases (28).

In this review, we outline the current knowledge about the involvement of acetate, propionate, and butyrate in microbiota-gut-brain interactions. We also highlight how the development of future treatments for CNS disorders can take advantage of the intimate and mutual interactions of the gut microbiota with the brain by exploring the role of SCFAs in the regulation of neuro-immunoendocrine function.

## The Microbiota-Gut-Brain Axis

The modulation of gut physiology by the CNS and its effects on gut function such as motility, secretion, blood flow, nociception, and immune function during neurological stressors are well-documented (17, 29, 30). Further, brain to gut signaling can directly affect the microbiota, either via immune system or gut functions such as motility, release of neurotransmitters and intestinal immune tone (12, 17, 21, 31). Comparatively, gut to CNS signaling has been studied for a short period, and the mechanisms underlying this crosstalk are starting to be understood (13, 32). It is noteworthy that several brain disorders have been linked to imbalances in the microbial composition of the gut (17, 19, 29, 33–37); however, whether these alterations in the microbiota are induced by brain signaling or whether brain dysfunction is driven by changes in the gut microbiota remains to be fully determined.

Although a more compelling causal relationship between altered gut microbial composition and brain dysfunction is still needed, it has been shown that disruption in the neuronal and microbial organization in prenatal and postnatal periods of mammalian development may lead to the onset of neurodevelopmental and other brain disorders later in life (9, 38–40). In a similar way, growing evidence has shown that alterations in maternal microbiome during pregnancy, such as use of antibiotics or probiotics (41, 42), variations in diet (43), immune activation (44, 45), and exposure to stress (46) can modulate the microbiome, neurodevelopment, and behavior of offspring in both rodents and humans (9, 29). Furthermore, delivery mode (47) and early-life occurrences such as feeding changes, infection, and antibiotics treatment (48, 49) have a huge effect on the gut microbiota composition with a long-term impact on brain and behavior (9, 29).

Under physiological conditions, activation of immune cells and production of cytokines can have a minor impact in the CNS. However, chronic systemic inflammation, mostly in the form of infections, has long been associated with behavioral alterations and cognitive dysfunction (50, 51). It is now widely known that peripheral insults that cause a systemic inflammatory response might affect ongoing inflammation in the CNS mainly by microglial activation, production of inflammatory molecules, as well as recruitment of peripheral immune cells into the brain, thus shaping a cerebral inflammatory milieu that may seriously affect neuronal function (50, 52, 53). Noteworthy, during gut pathologies with increased permeability of the intestinal barrier, the translocation of bacterial products can increase the production of cytokines and impact the blood-brain barrier (BBB), leading to more intense harmful effects (37). Further, it has already been shown that several bacterial strains can modify levels of neurotransmitter precursors in the gut lumen and even independently synthesize (or modulate the synthesis of) a number of neurotransmitters, including γ-aminobutyric acid (GABA), serotonin (5-HT), dopamine (DA), and noradrenaline (NA) (16–18). These neurotransmitters can potentially influence microglial activation and several cerebral functions (54). Additionally, the sympathetic branch of the autonomic nervous system is also involved in intestinal homeostasis and immune regulation (30). Conversely, the gut microbiota can interact with the CNS via gut modulation or directly via metabolites and endotoxin translocation from the lumen to the circulation (9, 17, 21). Possible signal transducers involved in the communication of the microbiota with the CNS include enterochromaffin cells, which can bind several microbial products and secrete serotonin into the lamina propria, increasing colonic and blood concentrations of 5-HT (55, 56). Gut-brain communication can also be achieved through vagus nerve signaling (57). Changes in enteric neuron activity perceived by the vagus nerve are essential for mediating satiety, stress, and mood (12, 58, 59). Given the close physical proximity, gut bacteria can interact with and activate the vagus nerve, thereby exerting effects upstream to the CNS. This notion is in full accordance with early studies showing that oral inoculation with pathogens or probiotics induces activation of the vagal sensory neurons that innervate the GI affecting the regulation of CNS functions, and this effect is absent in vagotomized mice (32, 58, 60). However, whether the vagus nerve is activated by physical interaction with bacteria or through soluble microbial components remain to be determined.

Finally, bacterial metabolic byproducts including SCFAs are often considered key candidate mediators of gut-brain communication, and altered SCFA production has been demonstrated in a variety of neuropathologies (19, 21, 33–35).

## Metabolism and Peripheral Effects of SCFAs

SCFAs are small organic monocarboxylic acids with a chain length of up to six carbons atoms and are the main products of the anaerobic fermentation of indigestible polysaccharides such as dietary fiber and resistant starch produced by the microbiota in the large intestine (61, 62). Comprised mostly of acetate (C2), propionate (C3), and butyrate (C4) (63, 64) in an approximate molar rate of 60:20:20, respectively (65), approximately 500–600 mmol of SCFAs are produced in the gut per day depending on the fiber content in the diet, microbiota composition, and gut transit time (66, 67). Although anaerobic fermentation of fibers is the largest source of SCFAs, acetate, propionate, and butyrate can also be produced from amino acid metabolism (68). However, less than 1% of the large intestine microbiota uses these metabolic pathways to produce SCFAs (69, 70). Protein fermentation usually takes place in the distal large intestine where carbohydrates are already depleted and also leads to the production of potentially toxic metabolites, such as ammonia, phenols, and sulfides, as well as unique branched-chain fatty acids (BCFA) (69, 71). Further, acetate produced from acetyl-CoA derived from glycolysis can also be transformed into butyrate by the enzyme butyryl-CoA:acetyl-CoA transferase (72, 73), and bovine milk fats also provide a source of butyrate (74).

Following their production, SCFAs are absorbed by colonocytes, mainly via H^+^-dependent or sodium-dependent monocarboxylate transporters (MCTs and SMCTs, respectively) (75). MCTs show different subtypes and expression patterns in different tissues. SCFAs that are not metabolized in the colonocytes are transported into the portal circulation and are used as an energy substrate for hepatocytes (76), except for acetate that is not oxidized in the liver (76). Therefore, only a minor fraction of colon-derived acetate, propionate, and butyrate reaches the systemic circulation and other tissues (65). In this context, it is important to note that most of the recent works regarding microbial-derived SCFA, mainly in human studies, use fecal concentrations as a proxy of the production in the colon (17, 19, 29, 33–37). Although it represents a valid approach, there are many potential sources of bias, such as intestinal transit and permeability, metabolite transportation, and sample handling (77). Thus, these drawbacks must be taken into account when concluding the effects of administered SCFAs, given that some experiments might be conducted under non-physiological conditions.

SCFAs improve the gut health through a number of local effects, ranging from maintenance of intestinal barrier integrity, mucus production, and protection against inflammation to reduction of the risk of colorectal cancer (78–81). Although a thorough comprehension of signaling triggered by SCFAs is still lacking, it is already known that SCFAs bind to G protein-coupled receptors (GPCRs). The best-studied SCFA receptors are GPR43 and GPR41, later renamed free fatty acid receptor (FFAR2) and FFAR3, as well as GPR109a/HCAR2 (hydrocarboxylic acid receptor) and GPR164, which are expressed in a vast array of cells, from the gastrointestinal mucosa to the immune and nervous systems (82, 83). The effects of activation of these receptors differ greatly depending on the cell on which they are expressed. For instance, binding of SCFAs to their receptors on enteroendocrine cells results in stimulated secretion of glucagon-like peptide 1 (GLP-1) and peptide YY (PYY) (84), while signaling in β-pancreatic cells leads to increased insulin secretion (85).

Another mechanism by which SCFAs regulate systemic functions is through the inhibition of histone deacetylase (HDAC) activity, thus promoting the acetylation of lysine residues present in nucleosomal histones throughout various cell populations (20). This intracellular signaling mechanism has been found in both the gut and associated immune tissue (86), as well as in the peripheral nervous system and CNS (20).

Although only a minor fraction of colon-derived SCFAs reaches the systemic circulation and other tissues, their effects on different organ and systems have recently been widely outlined. One of the best-documented effects of SCFAs is on the immune system since butyrate is capable of inducing Treg differentiation and controlling inflammation (17, 23, 24, 87). Although fine-tuning of the gut immune response to the microbiota is still a matter of debate, microbiota metabolites are capable of alleviating or worsening gut conditions such as inflammatory bowel disease (88). Effects on brown adipose tissue activation (89), regulation of liver mitochondrial function (90), whole-body energy homeostasis (91), and control of appetite (89) and sleep (10) have been attributed to all SCFAs. Further, the influence of the microbiota and the effects of SCFAs on the CNS have been a matter of intense debate in the last few years.

## SCFAs and the Brain

**Figure 1:**
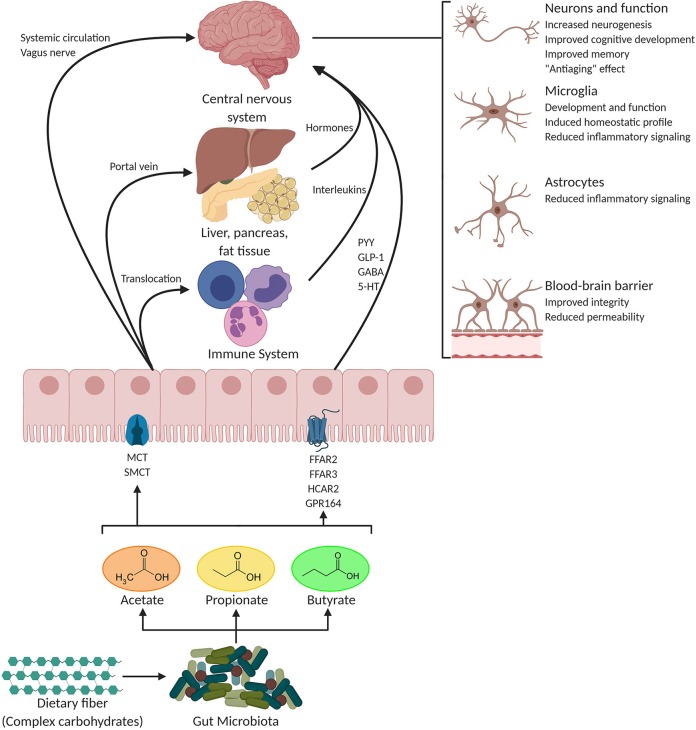
Potential pathways through which SCFAs influence gut-brain communication. Short-chain fatty acids (SCFAs) are the main metabolites produced by the microbiota in the large intestine through the anaerobic fermentation of indigestible polysaccharides such as dietary fiber and resistant starch. SCFAs might influence gut-brain communication and brain function directly or indirectly. Following their production, SCFAs are absorbed by colonocytes, mainly via H^+^-dependent monocarboxylate transporters (MCTs) or sodium-dependent monocarboxylate transporters (SMCTs). Through binding to G protein-coupled receptors (GPCRs) such as free fatty acid receptor 2 and 3 (FFAR2 and FFAR3), as well as GPR109a/HCAR2 (hydrocarboxylic acid receptor) and GPR164 or by inhibiting histone deacetylases, SCFAs influence intestinal mucosal immunity, and barrier integrity and function. SCFA interaction with their receptors on enteroendocrine cells promotes indirect signaling to the brain via the systemic circulation or vagal pathways by inducing the secretion of gut hormones such as glucagon-like peptide 1 (GLP1) and peptide YY (PYY), as well as γ-aminobutyric acid (GABA), and serotonin (5-HT). Colon-derived SCFAs reaches the systemic circulation and other tissues, leading to brown adipose tissue activation, regulation of liver mitochondrial function, increased insulin secretion by β-pancreatic cells, and whole-body energy homeostasis. Peripherally, SCFAs influence systemic inflammation mainly by inducing T regulatory cells (Treg) differentiation and by regulating the secretion of interleukins. SCFAs can cross the blood-brain barrier (BBB) via monocarboxylate transporters located on endothelial cells and influence BBB integrity by upregulating the expression of tight junction proteins. Finally, in the central nervous system (CNS) SCFAs also influence neuroinflammation by affecting glial cell morphology and function as well as by modulating the levels of neurotrophic factors, increasing neurogenesis, contributing to the biosynthesis of serotonin, and improving neuronal homeostasis and function. Together, the interaction of SCFAs with these gut-brain pathways can directly or indirectly affect emotion, cognition, and pathophysiology of brain disorders. Figure of this review was created with BioRender (https://biorender.com/).

In addition to exerting local effects in the colon and in the peripheral tissues, SCFAs are speculated to play a pivotal role in microbiota-gut-brain crosstalk (Figure 1). The abundant expression of MCTs in endothelial cells (75, 92) might facilitate crossing of the BBB by SCFAs since brain uptake of SCFAs has previously been demonstrated in rats following injection of ^14^C-SCFAs into the carotid artery (93). Although studies on physiological concentrations of SCFAs in the brain are scarce, all three metabolites are detectable in the human cerebrospinal fluid (CSF), typically in the range of 0–171 μM for acetate, 0–6 μM for propionate, and 0–2.8 μM for butyrate (94). An average concentration of 17.0 pmol/mg of tissue for butyrate and 18.8 pmol/mg of tissue for propionate in the human brain was reported (95). Furthermore, the levels of butyrate in the brain of mice supplemented with live *Clostridium butyricum* reached a range from 0.4 to 0.7 μmol/g, which was about an order of magnitude higher than concentrations reported in peripheral blood (96, 97). In addition to crossing BBB, SCFAs seem to play an important role in maintaining its integrity, which is tightly associated with controlled passage of molecules and nutrients from the circulation to the brain, playing a central role in brain development and the preservation of CNS homeostasis. Supporting the notion that SCFAs regulate the BBB function, germ-free (GF) mice show reduced expression of tight junction proteins such as claudin and occludin, leading to increased permeability of the BBB from intrauterine life to adulthood (98). Furthermore, recolonization of these adult mice with a complex microbiota or monocolonization with SCFA-producing bacterial strains recovers the integrity of the BBB (98). Similarly, treatment of an *in vitro* model of cerebrovascular endothelial cells with propionate attenuates the permeabilizing effects of exposure to lipopolysaccharide (LPS) (99).

Accumulating evidence suggests that SCFAs that cross into the CNS have neuroactive properties. Although the precise mechanisms involved in the action of SCFAs on the CNS remain largely unknown, a multitude of animal studies have shown that they exert widespread influence on key neurological and behavioral processes and may be involved in critical phases of neurodevelopmental and neurodegenerative disorders (17, 21, 29, 36, 100).

### SCFAs and Microglia

The development of the nervous system is marked by the sculpting of the neuronal networks shaping the functional neural circuitry that is critical for normal cognitive, emotional, and social domains. In this context, glial cells, especially microglial cells, have been increasingly recognized to play a critical role in the elimination of excess or unnecessary synaptic connections, which is necessary for the maturation and refinement of circuits and connections in the nervous system (101, 102). Therefore, control of innate immune function in the CNS is critical for brain development, and the gut microbiota seems to play a pivotal role in the development and functionality of the immune system in the CNS. The results reported by Erny and collaborators shed light on how the microbiota might influence microglial maturation and function (6). While microglia from specific pathogen-free (SPF) mice shows normal maturation and function, non-colonized young GF mice exhibit stunted microglia under homeostatic conditions. It is noteworthy that the oral application of a mixture of the three major SCFAs acetate, propionate, and butyrate was sufficient to drive maturation of microglia in GF mice (6). Although the mechanisms involved in the control of maturation and function of microglia by SCFAs remain to be determined, the activation of FFAR2 could be conceivable since FFAR2-deficient mice displayed microglia reminiscent of those found in GF mice (103).

Neuroinflammation is also an important process shaping brain function. Similar to observations in GF mice, perturbations of the gut microbiota by antibiotics systemically produce altered immune responses in experimental models, notably toward a pro-inflammatory profile (6). This is also true in the CNS, which becomes more prone to extreme inflammatory responses when the microbiota is depleted by antibiotics early in life (104). It was shown that antibiotic-induced perturbations in gut microbial diversity influence neuroinflammation with altered microglial morphology (105–107). On the other hand, several studies have reported that sodium butyrate is capable of decreasing microglial activation and pro-inflammatory cytokines secretion (108–110). Also, butyrate treatment *in vitro* and *in vivo* induces morphological and functional changes in the microglia toward a homeostatic profile and inhibits LPS-induced pro-inflammatory modifications (109) and depression-like behavior (110). Likewise, acetate treatment of microglia primary culture has been shown to reduce inflammatory signaling through reduced IL-1β, IL-6, and TNF-α expression and p38 MAPK, JNK, and NF-κB phosphorylation (111). Similarly, acetate was also able to modulate inflammatory cytokines and signaling pathways in astrocyte primary culture (112). Although the precise signaling involved in the effects of SCFAs on microglia remain unveiled, inhibition of HDACs, which results in epigenetically regulated gene expression, has been considered the main effector mechanism triggered by SCFAs (113). In this way, histone acetylation seems to modulate glial cells in an anti-inflammatory and neuroprotective manner. Therefore, taking into account the role of microglia in shaping neuronal networks and the influence of the microbiota on this process, SCFAs might provide new methods to modulate the brain immunity disruption underlying neurodevelopmental and neurodegenerative disorders.

### SCFAs and Neurons

Apart from providing the cells with energy and affecting microglia maturation, these microbial metabolites also seem to influence neuronal function. It was described that SCFAs may modulate the levels of neurotransmitters and neurotrophic factors. Acetate has previously been shown to alter the levels of the neurotransmitters glutamate, glutamine and GABA in the hypothalamus and increase anorexigenic neuropeptide expression (114). Propionate and butyrate exert an influence on the intracellular potassium level, which implies the involvement of SCFAs in the operation of cell signaling systems (115). In particular, these SCFAs regulate the expression levels of tryptophan 5-hydroxylase 1, the enzyme involved in synthesis of serotonin, and tyrosine hydroxylase, which is involved in a rate-limiting step in the biosynthesis of dopamine, noradrenaline and adrenaline; therefore, producing an effect on brain neurochemistry (21, 55, 56, 116, 117). Antibiotic depletion of the microbiota also results in hippocampal neurogenesis and memory impairments, which can be partially recovered by the reconstitution of specific SPF microbiota and completely recovered by probiotic treatment or exercise (118). This cognitive deficit might be associated with changes in the expression of cognition-relevant signaling molecules such as brain-derived neurotrophic factor (BDNF), N-methyl-D-aspartate receptor subunit 2B, serotonin transporter and neuropeptide Y system (119).

Neurotrophic factors, such as nerve growth factor (NGF), glial cell line-derived neurotrophic factor (GDNF), and BDNF that regulate the growth, survival and differentiation of neurons and synapses in the CNS also play important parts in learning and memory and in a range of brain disorders have been also shown to be modulated by SCFAs (120–123). BDNF expression, neurogenesis, and neural proliferation in rodents (124–126), as well as facilitation of long-term memory consolidation, were stimulated by sodium butyrate (127). Further, physiological levels of all three SCFAs were shown to increase the growth rate of human neural progenitor cells and induce more cells to undergo mitosis (128), affording some hints of how SCFAs could regulate early neural system development. Further, SCFAs show effects on several neural functions, such as enhancing sleep (10), suppressing the activity of orexigenic neurons that express neuropeptide Y in the hypothalamus (89), and modulating the signaling triggered by the ghrelin receptor (129), contributing to circadian rhythm and appetite control. The seeking for mechanism involved in the modulation of neuronal function by SCFAs has unveiled that some of these effects are likely mediated by the activation of GPR41/GPR43 receptors. Other SCFA effects, especially of propionate and butyrate, are mediated through their HDAC inhibitory activity (108, 116).

Because of the similarity of SCFAs with the ketone bodies aceto-acetate and β-hydroxybutyrate (BHB), studies have been conducted to elucidate their role during fasting. Accordingly, fasting has been shown to sharply influence the gene regulation and protein expression of several MCTs, which alters the uptake of SCFAs in the gut and their transport to the brain (130). The regulation of the transporter is likely related to the direction of energy supplies to tissues during fasting. Moreover, Miletta and colleagues found that butyrate enhances growth hormone (GH) secretion in pituitary cells via GPR41/43 activation and intracellular accumulation of Ca^2+^ (131). This leads to the hypothesis that butyrate acts as a secondary mediator of metabolic adaptations of GH during fasting, which mainly include increased lipolysis and protein retention.

In summary, SCFAs might directly influence the brain by reinforcing BBB integrity, modulating neurotransmission, influencing levels of neurotrophic factors and promoting memory consolidation. However, further studies are needed to understand the precise mechanisms involved in these neuroactive effects.

## SCFAs and Brain Disorders

The synthesis of new proteins is necessary for long-term changes in synaptic plasticity and learning (132–134). In this context, learning and long-term memory formation are improved by enhanced histone acetylation (135), which could be improved by HDAC inhibitors (HDACi). Given the HDAC inhibition property of SCFAs, several animal studies have focused mainly on the use of butyrate to elevate histone acetylation in the brain during a critical phase of memory formation. These studies have reported an enhancement of long-term potentiation (LTP) and contextual fear memory induced by HDAC inhibition (124, 127, 136, 137), pointing out enteric SCFAs as a promising learning and memory modulators. Therefore, the discovery that the microbiota can influence brain physiology has led to a plethora of experiments involving neurological disorders. The central hypothesis is supported by experimental and clinical evidence that the microbiota is altered in such diseases, which aggravates the condition, and/or its modulation might prevent or improve the development and progression of CNS pathologies (17, 19, 29, 33–37). Interestingly, several studies have found that the gut microbiome composition and, consequently, metabolome are altered in many brain disorders (138–142). Despite the knowledge that microbiota-gut-brain communication can theoretically occur through multiple systems (including the autonomic nervous system, enteric nervous system, neuroendocrine system, and immune system), increased evidence supports a potential key role of SCFAs in gut-brain axis signaling, and alterations in this signaling might underpin CNS disturbances ranging from neurodevelopmental disorders to neurodegenerative diseases.

### SCFAs and Autism Spectrum Disorder

Characterized by behavioral symptoms including communication deficits, repetitive behaviors, and sensitivity to environmental changes, ASD comprises an array of neurodevelopmental disorders (143). Imbalances in the microbial composition of the gut are present in ASD. Support for this notion originates from animal studies and clinical evidence. However, the role of SCFAs in ASD is still controversial. Recently, Sharon and collaborators showed that microbiota transplantation from human ASD donors into mice could transfer ASD-relevant behavioral deficits (27). Although Sharon and coworkers did not evaluate the alteration in SCFAs, children with ASD have been previously reported to have both lower (144) and higher (33) fecal SCFA levels than controls. Interestingly, Wang and coworkers found similar proportions of specific SCFA and protein fermentation metabolites when comparing children with ASD with controls, even though the groups were controlled for gastrointestinal abnormalities, macronutrients intake and usage of probiotics, prebiotics, and antibiotics (33). However, neither of the previous studies performed a comprehensive evaluation of microbiota ecology.

In line with these findings, the microbiota has been suggested to affect the occurrence and severity of the disease through an increase in propionate-producing bacteria and a decrease in butyrate-producing bacteria (145, 146). The study conducted by Finegold and coworkers also found several pathobionts increased in the stool of ASD affected children such as *Proteobacteria* and hydrogen sulfide producing *Desulfovibrio*, raising a question for the causality of microbial metabolites unbalance (145, 146). Further, propionate-induced autism has become a validated animal model to study the disease. Administering high amounts of propionate through subcutaneous, intragastric, intraperitoneal, or intracerebroventricular routes to rodents has been suggested to induce high levels of microglia activation, neurotoxic cytokine production, genetic expression alterations, abnormal hippocampal histology, and abnormal neurobehaviors, such as repetitive actions and impaired social interaction (147). On the other hand, butyrate appears to have a beneficial effect on social and repetitive behavior in the BTBR mouse model, a strain-based ASD-like model (148). Epigenetic changes led to enhanced transcription of inhibitory neurotransmitter pathways in the frontal cortex, especially through HDAC inhibition (148). As described above, improvement of BBB impermeability by butyrate may be another mechanism through which butyrate can revert abnormalities in propionic acid-induced autism-like disorder (143). This evidence points to the importance of balance of a microbiota but also highlights the difficulty in drawing conclusions on the role of SCFAs in ASD and the need for more research in patients with ASD.

### SCFAs and Mood Disorders

Despite the complex pathophysiology of mood disorders, several studies have indicated the participation of the gut microbiota in the severity of these diseases. Major depression is one of the most common mood disorders, seriously impairing the quality of life of patients and is one of the leading causes of social disability. Untreated depression is associated with an increased risk of morbidity and mortality, including suicide. Monoamine deficiency (149) and neurogenesis disruption (150) are two predominant theories underpinning depression. Furthermore, it has been shown that inflammation biomarkers are increased among patients with depression, and pro-inflammatory cytokines play an important role in the physiopathology of the disease (150). The importance of the microbiota in depression is supported by findings that the levels of SCFAs are decreased in a naturally occurring non-human primate model of depression (26). In line with these findings, clinical evidence has shown that fecal SCFA concentrations are lower in patients with depression than in controls (35, 151). Moreover, current knowledge shows that butyrate possesses an antidepressant-like effect that reverses behavioral alterations in mouse models, such as low energy (126, 152), anhedonia (153), and cognitive and sociability impairments (154). Therefore, taking into account the anti-inflammatory property of SCFAs, dysbiosis followed by decreased levels of these metabolites could play a role in the inflammation process related to the development of depression.

Studies on chronic psychosocial stress have also shown a possible application for prebiotics (154) and SCFAs (8) in reverting sociability impairment while also reducing stress-induced corticosterone release. Sodium butyrate has been shown to be capable of reversing behavioral hyperactivity (155) and depressive-like and manic-like behaviors in rats (156). There is also evidence for butyrate's antimanic effect on a rat model of bipolar disorder induced by intracerebroventricular administration of ouabain (157). Contrarily, a microbiome study in schizophrenic patients at risk of developing psychosis reported enriched *Clostridiales, Prevotella*, and *Lactobacillus ruminis* and predicted increased SCFA production (141). However, the study did not perform direct measurement of the metabolites and further research to confirm whether it is a case of SCFA overproduction or a specific metabolite unbalance is needed.

### SCFAs and Alzheimer's Disease

Accumulating evidence has demonstrated that key neuropathological processes underlying AD might also be modulated by SCFAs (25, 34, 158, 159). Characterized by progressive cognitive impairment, AD is the most common form of dementia (160). Given that AD has a complex pathology and that therapies that effectively halt the disease progression are still lacking, recent studies have focused on environmental components and diet-based possible prevention strategies by using transgenic animal models (161, 162). In this context, several studies have established the benefits of a healthy microbiome on slowing AD and the correlation of dysbiosis with disease progression (7, 138, 163). Consistent with this notion, a study by Zhang and coworkers showed that the microbiota composition and diversity were perturbed and the level of SCFAs was reduced in AD mice, predicting alterations in more than 30 metabolic pathways, which may be associated with amyloid deposition and ultrastructural abnormalities in the APP/PS1 mouse model (25).

It is worth noting that SCFAs interfere with protein-protein interactions between amyloid-β peptides (Aβ), thereby disrupting their assembly into neurotoxic oligomers (34), the main toxins responsible for synapse dysfunction and cognitive deficits in AD (164). Given the close relation between gut dysbiosis and brain dysfunction, fecal microbiota transplantation (FMT) has been considered a promising therapeutic approach for the reestablishment of a healthy gut microbial community and has been shown to have beneficial effects on a plethora of diseases, including AD. Supporting this hypothesis, APP/PS1 mice exhibited significantly relieved cognitive deficits, Aβ accumulation, synaptic dysfunction, and neuroinflammation, mainly by the microglia, after FMT from healthy wild-type mice (165). These protective effects may be related to reversal of changes in the gut microbiota and SCFAs.

Oral bacteriotherapy through probiotic administration has become a potential treatment option for neurodegenerative diseases such as AD. Accordingly, the 3xTg mouse model of AD treated with probiotics in the early stage showed a promising reduction of inflammatory cytokines and decreased cognitive decline associated with reduced brain damage and Aβ aggregate accumulation (166). Moreover, other studies have shown beneficial effects of butyrate and probiotic treatment on cognition and memory in a D-galactose model of aging, a condition known to correlate with AD occurrence and progression (137, 167). The model consists of a long term administration of D-galactose, which can readily be metabolized but eventually leads to an overproduction of reactive oxygen species, thus causing genetic and cell damage impairing cognition (137, 168). Finally, through HDAC inhibition, butyrate administration recovered memory function and increased expression of genes implicated in associative learning in the APP/PS1 mouse model of AD (158).

### SCFAs and Parkinson's Disease

SCFAs play a controversial role in Parkinson's disease (PD), a synucleinopathy and a multifactorial disorder with strong environmental influence characterized by tremors, muscle rigidity, bradykinesia, and impaired gait (169). Aggregation of the protein α-synuclein (αSyn) is thought to be the main pathogenic event in PD, which primarily affects dopaminergic neurons (169). Most PD patients also present gastrointestinal manifestations due to disturbances of the enteric nervous system. Hence, there has been great interest in the relationship between the gut microbiota and the development of the disease. Accordingly, sequencing of the microbiota of fecal samples from PD patients revealed reduced populations of *Bacteroidetes* and *Prevotellaceae* in contrast to increased *Enterobacteriaceae* and reduced production of SCFAs when compared to matched controls (139). However, the presence of gut microbes is necessary to elicit pathophysiological alterations in a mouse model of αSyn overexpression, because elimination of the gut microbiota with antibiotics ameliorated the condition (169). In contrast, FMT from PD patient donors worsens disease progression suggesting the presence of specific disease-promoting microbes (169). Accordingly, Li and colleagues confirmed that PD patients suffer alterations in the microbiota that correlate with disease progression, as there is a continuous decrease in fiber-degrading bacterial strains and an increase in pathobionts (170). This conversion probably leads to a decrease in SCFA production and an increase in endotoxin and neurotoxin production (170). Supporting this hypothesis, growing evidence has shown that FMT from healthy donors (171) as well as butyrate administration in animal models of PD improves motor impairment and dopamine deficiency (172–175).

### SCFAs and Sclerosis

Multiple sclerosis (MS) is a neurodegenerative T-cell-mediated autoimmune disease of the CNS that mainly affects the myelin sheath around motor neurons. Among its etiological factors, the imbalance between pro and anti-inflammatory cells in the immune system seems to play an important role, which is highly affected by the gut microbiota composition and can be aggravated by dysbiosis (104, 176, 177). Given that SCFAs, mainly butyrate, are capable of inducing Treg polarization, modulation of the gut microbiota toward increased production of these metabolites could be an interesting therapeutic approach to MS. In fact, it is noteworthy that oral administration of SCFAs ameliorated the disease severity of experimental autoimmune encephalomyelitis (EAE), an animal model of MS (87, 178). Specifically, acetate supplementation is able to induce increased acetyl-CoA metabolism, which increases histone acetylation, resulting in preserved spinal cord lipid content and essentially preventing the onset of clinical symptoms of EAE (179). Furthermore, treatment with butyrate suppresses demyelination and enhances remyelination through oligodendrocyte maturation and differentiation (180).

Efforts to modify the course of amyotrophic lateral sclerosis (ALS) a disease that affects motor neurons but also involves a stronger genetic basis that leads to the premature death of those cells, has focused on the gut microbiota composition and its circulating metabolites (181). A comparative study conducted in human patients showed an elevated relative abundance of pathobionts compared to bacterial strains related to beneficial metabolism function (142). Another study found that transgenic ALS model mice had worse disease progression when raised under antibiotic treatment or GF conditions and identified several bacterial strains correlated with ameliorated or aggravated disease progression. A small assessment of the human microbiome/metabolite configuration was also conducted for comparison (181).

### SCFAs and Metabolic Disorders

Much speculation currently surrounds the possible involvement of the gut microbiota in metabolic disorders such as type 2 diabetes and obesity. Compelling evidence have shown that the composition of the gut microbiota is altered in animal models of obesity and subjects with prediabetes or type 2 diabetes compared with controls (182–186). Despite differences in the identification of specific microbiome features responsible for these effects, a shift in the microbiome composition away from species able to produce butyrate was one consistent finding in type 2 diabetes subjects (187). Further, epidemiological and experimental studies have demonstrated that increased intake of dietary fiber reduces the risk for developing metabolic diseases (188–190), possibly by changing gut microbiome composition and diversity with increased production of SCFAs (187–189).

Animal studies suggest that SCFAs have an important role in the prevention and treatment of obesity-associated insulin resistance (89, 114, 191, 192). Mechanisms involved in the effects of SCFAs, mainly propionate and butyrate, in the brain responsible for controlling metabolic disorders include the activation of FFAR2 and FFAR3 receptors (91). It was shown that activation of these receptors leads to suppression of the activity of orexigenic neurons that express neuropeptide Y in the hypothalamus (89), and the modulation of the signaling mediate by the ghrelin receptor (129), contributing to circadian rhythm and appetite control. Studies in rodents show that the administration of prebiotics that influences a shift in the gut microbiome toward increased production of butyrate has beneficial effects associated with higher levels of GLP-1 (193–195), as well as hypothalamic expression of pro-opiomelanocortin (196), thereby influencing the hunger-satiety cycle. Although limited, some of these results were confirmed in human *in vivo* studies, as showed that acute rectal infusions of sodium acetate and SCFA mixtures increased circulating concentrations of PYY in individuals who were overweight (197–199).

## Concluding Remarks

The gut microbiota has attracted considerable attention in recent years, putting it in the spotlight of biomedical research. Recent studies have suggested that an intestinal bacteria imbalance plays a role in the development of several disorders. The bidirectional communication that occurs between the microbiota and its mammalian host can be mediated through a variety of mechanisms, and it is clear that the biochemical messengers produced by the microbiota are an important facet of this crosstalk. Convincing evidence exists that SCFAs produced by the intestinal microbiota are involved in gastrointestinal physiology, immune function, host metabolism, and even in development and homeostasis of the CNS.

Although our understanding of microbiota-host interactions has considerably increased over recent years, there is still an unmet requirement for a deeper understanding of the complex microbiota-gut-brain communication. Furthermore, since most studies have been conducted in rodents, one must be cautious when translating the effects of SCFAs on humans. Given that SCFAs can regulate CNS processes through direct and indirect means and ultimately shape behavior and cognitive function, a thorough comprehension of how these metabolites participate in these complex gut-brain interactions may aid in developing novel therapeutic targets for treating CNS disorders. Further, through their effects on the development and maintenance of healthy brain function, these metabolites hold the potential for use as dietary interventions with a range of psychological functions.

## Author Contributions

YS, AB, and RF planned, researched, and wrote the manuscript.

### Conflict of Interest

The authors declare that the research was conducted in the absence of any commercial or financial relationships that could be construed as a potential conflict of interest.

## Abbreviations

Aβ: amyloid-β peptide
AD: Alzheimer's disease
ASD: autism spectrum disorder
αSyn: α-synuclein
BBB: blood-brain barrier
BDNF: brain-derived neurotrophic factor
BHB: β-hydroxybutyrate
CNS: central nervous system
DA: dopamine
EAE: experimental autoimmune encephalopathy
FFAR: free fatty acid receptor
FMT: fecal microbiota transplantation
GABA: γ-aminobutyric acid
GDNF: glial cell line-derived neurotrophic factor
GF: germ free
GH: growth hormone
GI: gastrointestinal (tract)
GLP-1: glucagon-like peptide 1
GOS: galacto-oligosaccharides
GPCR: G protein-coupled receptors
HCAR2: hydrocarboxylic acid receptor
HDACs: histone deacetylases
HDACi: HDAC inhibitor
HPA: hypothalamus-pituitary-adrenal
LPS: lipopolysaccharide
MCT: H^+^-coupled monocarboxylate transporter
MS: multiple sclerosis
NA: noradrenaline
NGF: nerve growth factor
PD: Parkinson's disease
PKCδ: protein kinase Cδ
PYY: peptide YY
5-HT: serotonin
SCFAs: short-chain fatty acids
SMCTs: sodium-coupled monocarboxylate transporters
SPF: specific pathogen-free
Tregs: T-regulatory lymphocytes.
